# Supplementary material for: Perceptual coupling and decoupling of the default mode network during mind-wandering and reading
Source: eLife. 2022 Mar 21;11:e74011. doi: 10.7554/eLife.74011 (PMC8937216; doi:10.7554/eLife.74011)
Supplement: Transparent reporting form [file elife-74011-transrepform1.docx]

***eLife’s* transparent reporting form**

We encourage authors to provide detailed information *within their submission* to facilitate the interpretation and replication of experiments. Authors can upload supporting documentation to indicate the use of appropriate reporting guidelines for health-related research (see [EQUATOR Network](http://www.equator-network.org/%20)), life science research (see the [BioSharing Information Resource](https://biosharing.org/)), or the [ARRIVE guidelines](http://www.plosbiology.org/article/info:doi/10.1371/journal.pbio.1000412) for reporting work involving animal research. Where applicable, authors should refer to any relevant reporting standards documents in this form.

If you have any questions, please consult our Journal Policies and/or contact us: [editorial@elifesciences.org](mailto:editorial@elifesciences.org).

**Sample-size estimation**

- You should state whether an appropriate sample size was computed when the study was being designed
- You should state the statistical method of sample size computation and any required assumptions
- If no explicit power analysis was used, you should describe how you decided what sample (replicate) size (number) to use

Please outline where this information can be found within the submission (e.g., sections or figure legends), or explain why this information doesn’t apply to your submission:

No explicit power analysis was performed to determine the sample size in each experiment, however, the present sample sizes for task-based fMRI data analysis in Experiment 1 (N=29) and for intrinsic connectivity (N=243) and individual differences in functional connectivity (N=69) is comparable to, or significantly exceeds, that of prior task-based or resting-state fMRI studies examining the neural basis of mind-wandering reading (e.g., Zhang et al., 2019, Scientific Reports; Smallwood et al., 2013, Frontiers in Human Neuroscience).

**Replicates**

- You should report how often each experiment was performed
- You should include a definition of biological versus technical replication
- The data obtained should be provided and sufficient information should be provided to indicate the number of independent biological and/or technical replicates
- If you encountered any outliers, you should describe how these were handled
- Criteria for exclusion/inclusion of data should be clearly stated
- High-throughput sequence data should be uploaded before submission, with a private link for reviewers provided (these are available from both GEO and ArrayExpress)

Please outline where this information can be found within the submission (e.g., sections or figure legends), or explain why this information doesn’t apply to your submission:

1. In Experiment 1, each participant was asked to either focus on recalling personal memories with or without conflict from sentence inputs, or read factual sentences for comprehension with or without conflict from autobiographical memory. With each situation contained 24 individual trials, and was performed 29 times across 29 participants. This task is detailed described in the ***Task Procedure for Reading and Autobiographical memory task*** section, and is visualized in Figure 1.

In Experiment 2, another 313 participants were asked to finish a 9-minute resting-state scanning, during which they were asked to focus on a fixation presented in the centre of the screen. Additionally, for the participants in the individual difference dataset, they were also required to finish a behavioural mind-wandering reading assessment outside the scanner. Details of these data collection are also reported in the **Methods** section.

2. The details of exclusion/inclusion of data can be found in ***Participant****s* section: *One participant was excluded from this analysis due to excessive head motion (i.e., mean head motion > .4 mm)*;

3. For the potential outliers in the brain data, for task-based fMRI analysis in Experiment 1, a motion scrubbing (using the fsl_motion_outliers tool) was applied to exclude volumes that exceeded a framewise displacement threshold of 0.9 mm; instead, the confound effect from motion outliers was removed using the implemented anatomical CompCor approach in resting-state data analysis.

*For the outliers in behavioural assessment,* prior to data analysis, all behavioural variables were z-transformed and outliers more than 2.5 standard deviations above or below the mean were imputed with the cut-off value.

The details of how the outliers were handled can be found in ***Methods*** section.

4. We have uploaded our Neuroimaging data at the group-level in Neurovault, this information can be found in Data availability section: Neuroimaging data at the group level are openly available in Neurovault at <https://neurovault.org/collections/9432/>.

Other information is not applicable to the fMRI analyses performed in this study.

**Statistical reporting**

- Statistical analysis methods should be described and justified
- Raw data should be presented in figures whenever informative to do so (typically when N per group is less than 10)
- For each experiment, you should identify the statistical tests used, exact values of N, definitions of center, methods of multiple test correction, and dispersion and precision measures (e.g., mean, median, SD, SEM, confidence intervals; and, for the major substantive results, a measure of effect size (e.g., Pearson's r, Cohen's d)
- Report exact p-values wherever possible alongside the summary statistics and 95% confidence intervals. These should be reported for all key questions and not only when the p-value is less than 0.05.

Please outline where this information can be found within the submission (e.g., sections or figure legends), or explain why this information doesn’t apply to your submission:

1. The statistical tests used were reported using text and we reported statistics with degrees of freedom when applicable. Results were reported using mean ± SEM (e.g., in Figure 1), exact *p*-values, and effect size of *η_p_^2^* values when applicable (e.g., behavioural results in Experiment 1).

2. Bonferroni correction was also applied in individual differences in mind-wandering analysis, please see ***Analysis of resting-state fMRI data in Experiment 2:*** Bonferroni correction was applied to account for the fact that we included two models, the *p*-value consequently accepted as significant was *p* < 0.025.

For the statistical behaviour-brain correlation analysis in mind-wandering reading individual difference analysis, we reported exact coefficient *r*-values, *p*-values and 95% confidence intervals (i.e., in Figure 4).

All statistical analysis methods are described in the ‘Results’ and ‘Methods’ sections.

(For large datasets, or papers with a very large number of statistical tests, you may upload a single table file with tests, Ns, etc., with reference to sections in the manuscript.)

**Group allocation**

- Indicate how samples were allocated into experimental groups (in the case of clinical studies, please specify allocation to treatment method); if randomization was used, please also state if restricted randomization was applied
- Indicate if masking was used during group allocation, data collection and/or data analysis

Please outline where this information can be found within the submission (e.g., sections or figure legends), or explain why this information doesn’t apply to your submission:

This study examines the underlying neurocognitive mechanism of daily experience of mind-wandering while reading using healthy participants, and therefore no group allocations were made. The details of exclusion/inclusion of participants can be found in the **Methods** section (i.e. Participants).

**Additional data files (“source data”)**

- We encourage you to upload relevant additional data files, such as numerical data that are represented as a graph in a figure, or as a summary table
- Where provided, these should be in the most useful format, and they can be uploaded as “Source data” files linked to a main figure or table
- Include model definition files including the full list of parameters used
- Include code used for data analysis (e.g., R, MatLab)
- Avoid stating that data files are “available upon request”

Please indicate the figures or tables for which source data files have been provided:

Group-level statistical maps and source data files for Neurosynth decoding analysis (i.e. unthresholded statistical maps) have been provided for Figures 2-4, which can be accessed in Neurovault at <https://neurovault.org/collections/9432/>. Source data for pie charts in Figure 2C, and relevant information has been added in the relevant figure legends.

The conditions of our ethical approval do not permit public archiving of the data because participants did not provide sufficient consent. Researchers who wish to access the data should contact the Research Ethics and Governance Committee of the York Neuroimaging Centre, University of York, or the corresponding authors. Data will be released to researchers when this is possible under the terms of the GDPR (General Data Protection Regulation).

In addition, materials for reading comprehension and memory recall task, and task code are available at <https://osf.io/yvks7/>.
